# Toward engineered microbial chassis for growth–production balancing

**DOI:** 10.1016/j.synbio.2026.07.002

**Published:** 2026-08-06

**Authors:** Huanghui Xia, Bingmei Wang, Feng Qi, Jianzhong Huang

**Affiliations:** a College of Life Sciences, Fujian Normal University, Fuzhou, China; b Engineering Research Center of Industrial Microbiology of Ministry of Education, Fujian Normal University, Fuzhou, China

**Keywords:** Microbial cell factories, Growth–production trade-off, Microbial chassis, Systems metabolic engineering, Dynamic metabolic control, Spatial reorganization, AI-Enabled biomanufacturing

## Abstract

The inherent conflict between cellular growth and product synthesis, arising from limited resource allocation and metabolic burden, fundamentally constrains the performance of microbial chassis. Traditional strategies that focus solely on flux intensification or knockout of competing pathways often face diminishing returns and may compromise cellular fitness, motivating the exploration of alternative engineering paradigms. Emerging approaches aim to modulate the growth–production trade-off across temporal, network-level, and spatial dimensions, thereby reframing this trade-off from a fixed physiological barrier into a tunable design parameter. This review systematically summarizes four complementary strategies: temporal decoupling via dynamic genetic circuits and phase-segregated cultivation; network-level metabolic resource optimization through redistribution of carbon flux, cofactors, and energy; spatial reorganization using enzyme colocalization, organelle compartmentalization, and modular microbial consortia; and digital and intelligent control frameworks that integrate real-time sensing, artificial intelligence (AI)-based prediction, and digital twin–enabled closed-loop optimization. The key insight is that decoupling does not eliminate competition but rather reschedules, redistributes, or relocalizes it, thereby achieving high production without sacrificing cellular robustness. Collectively, these multi-dimensional strategies transform the growth–production trade-off into a programmable engineering variable. Future efforts should focus on simplifying dynamic circuits, improving long-term genetic stability under industrial conditions, developing tools for non-model hosts, and engineering generally recognized as safe (GRAS) organisms with predictable trade-off management, paving the way for next-generation microbial cell factories that are both productive and resilient.

## Graphical abstract

**Figure:**
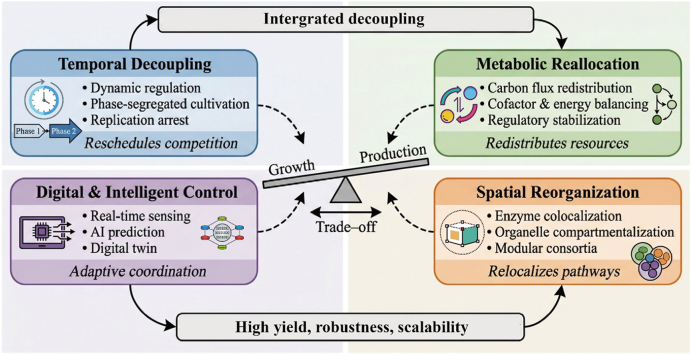

## 1 Introduction

Microbial metabolic engineering enables the reprogramming of cells into production platforms for fuels, materials, and high-value chemicals [1]. Despite substantial advances in systems metabolic engineering and synthetic biology, achieving high product titers while maintaining robust cellular growth remains a persistent challenge across diverse hosts and biosynthetic pathways [2]. A comprehensive evaluation of hundreds of target chemicals across multiple industrial microorganisms has shown that further improvements based solely on flux intensification frequently reach a plateau [3]. This observation reflects an intrinsic limitation arising from finite cellular resources [4].

The growth–production trade-off originates from the constrained allocation of intracellular resources and the tightly coupled organization of metabolic and regulatory networks. Central metabolism must distribute carbon, energy, and reduce equivalents between biomass formation and product synthesis. At the same time, heterologous pathway expression imposes additional burdens through enzyme overexpression, intermediate accumulation, and metabolic imbalance [5]. These constraints are further amplified by product and intermediate toxicity, which disrupt membrane integrity, redox homeostasis, and enzymatic activity [6,7]. Weak acids and alcohols exemplify such effects by increasing maintenance energy demand and impairing cellular physiology [[8], [9], [10]]. Integrative omics approaches have further revealed system-wide responses associated with toxic stress and metabolic perturbation [11]. These findings indicate that the growth–production trade-off should not be interpreted as a localized metabolic bottleneck. Instead, it represents a system-level constraint emerging from the coordination of resource allocation, network topology, and cellular homeostasis. Conventional strategies based on constitutive pathway strengthening or elimination of competing reactions often fail to overcome this constraint and may compromise long-term stability [12].

Recent efforts have therefore shifted toward multi-dimensional regulation that reorganizes metabolic functions across time, network structure, and spatial organization [13,14]. Temporal strategies exploit growth phase-dependent metabolic states to separate biomass accumulation from product synthesis [15]. Network-level metabolic resource optimization redistributes carbon flux, redox balance, and energy supply to enhance intracellular efficiency while sustaining growth [16]. Spatial reorganization restructures metabolic functions through compartmentalization or division of labor, thereby reducing pathway interference and mitigating intermediate toxicity [17]. In addition to these three primary engineering dimensions, digital and intelligent control frameworks are emerging as an additional regulatory layer that enables predictive and adaptive coordination of cellular states and bioprocess conditions. Data-driven modeling, artificial intelligence (AI), and cyber–physical integration support dynamic optimization of metabolic activity and process performance under industrially relevant conditions [14,18]. These approaches extend metabolic engineering from empirical optimization toward model-guided design and real-time control.

Collectively, the growth–production trade-off should be viewed not as a fixed physiological barrier, but as a tunable engineering variable that can be modulated across multiple organizational layers. This perspective is particularly relevant to food and agricultural biomanufacturing, where high productivity must be achieved together with process robustness, safety, and Generally Recognized as Safe (GRAS) compatibility [19,20]. Temporal decoupling separates biomass accumulation from biosynthesis by controlling when production pathways are activated. Network-level metabolic resource optimization redistributes intracellular carbon, redox, energy, and regulatory resources to reduce competition between growth and synthesis. Spatial reorganization restructures metabolic topology through compartmentalization, enzyme colocalization, and division of labor to minimize pathway interference. Digital and intelligent control adds a predictive and adaptive regulatory layer that links cellular engineering with real-time bioprocess optimization.

**Fig. 1:**
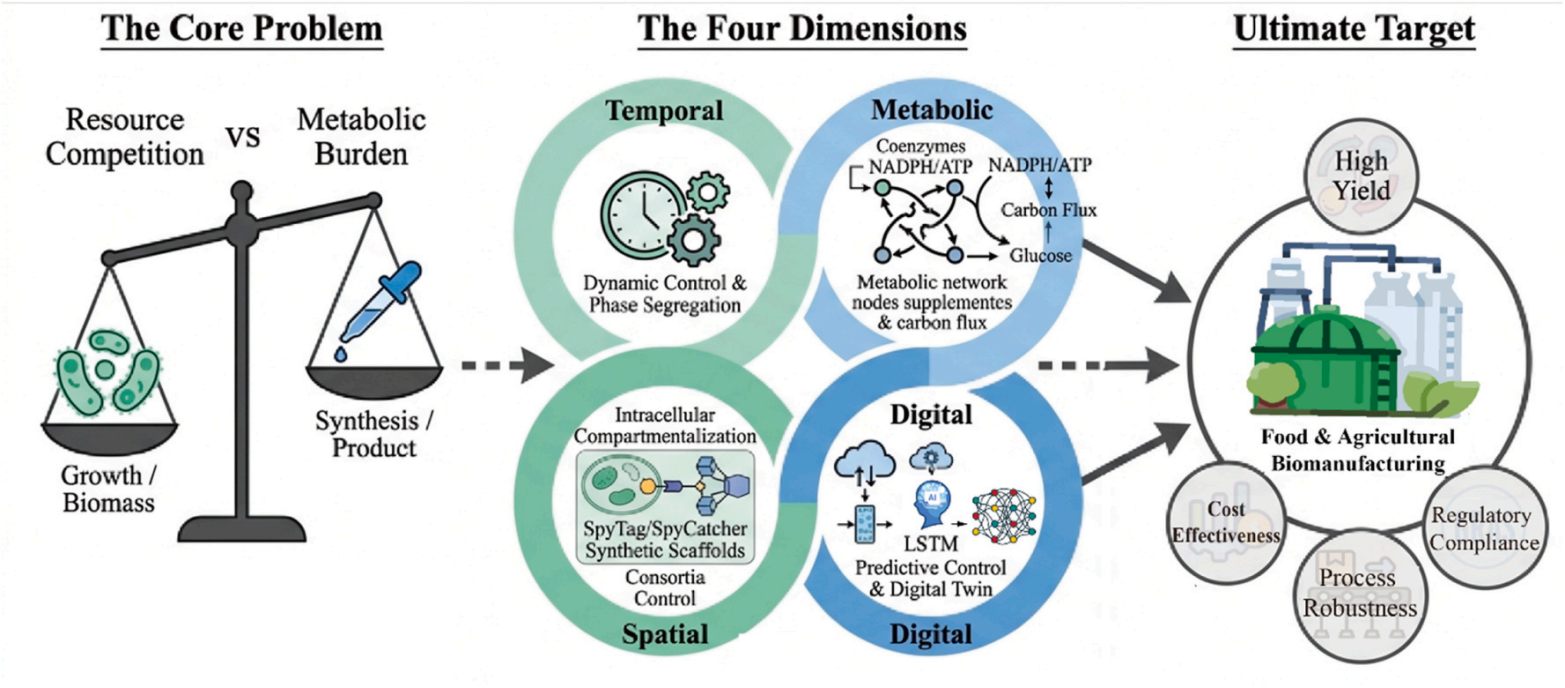
Multi-dimensional synergistic regulation strategies for advanced microbial cell factories in biomanufacturing.

In this review, we define a strategy as directly relevant to the growth–production trade-off only when it satisfies at least one of the following mechanistic criteria: it reduces competition for carbon precursors, alleviates ATP or redox cofactor limitation, lowers expression or toxicity burden during growth, separates incompatible metabolic states in time or space, or enables process-level control that prevents cells from entering low-productivity stress states. This criterion is used throughout the review to distinguish true trade-off-resolving strategies from general productivity-enhancing methods. Together, these dimensions provide a coherent framework for understanding how microbial chassis can be redesigned for robust and efficient biomanufacturing (Fig. 1).

## 2 Temporal decoupling of growth and production

Dynamic regulation represents one of the most direct strategies for temporally decoupling growth from production by enabling programmable control over the timing and intensity of pathway activation. Rather than constitutively expressing biosynthetic modules throughout the entire cultivation process, these systems introduce regulatory switches that activate or repress metabolic functions in accordance with cellular physiological states, thereby reducing unnecessary metabolic burden during early biomass accumulation and redirecting intracellular resources toward product formation at later stages.

### 2.1 Intracellular dynamic switches for autonomous temporal decoupling

**Fig. 2:**
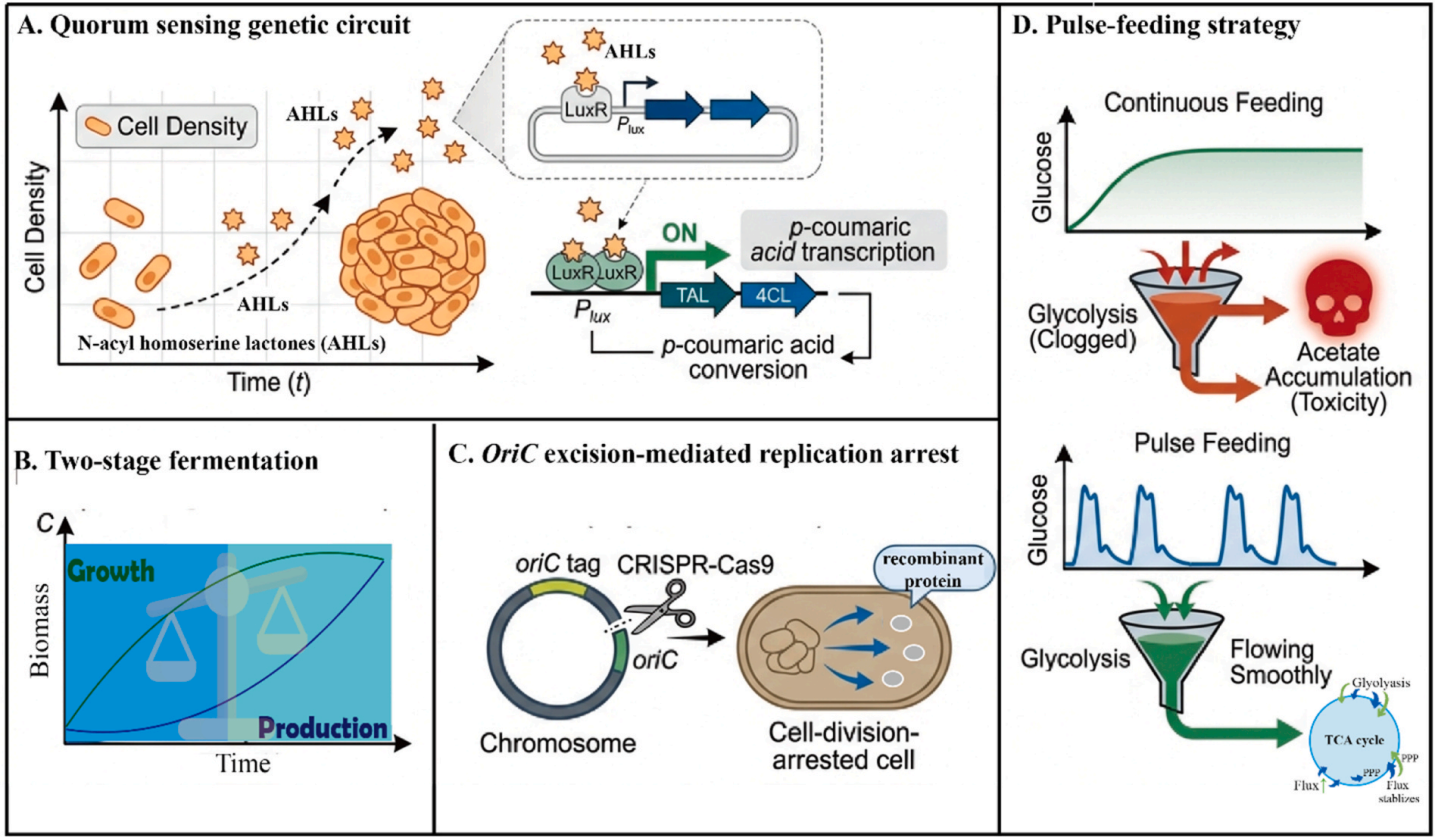
**Temporal decoupling strategies for balancing microbial growth and production.** A. Quorum sensing (QS)-based autonomous genetic circuit. Accumulation of N-acyl homoserine lactones (AHLs) activates cell-density-dependent pathway expression to switch cells from growth to production mode; B. Two-stage fermentation process. Temporally separates biomass accumulation and product synthesis through externally controlled cultivation phases; C. *OriC* excision-mediated replication arrest. CRISPR–Cas9 removes the chromosomal origin of replication to halt cell division while maintaining metabolic activity for enhanced product synthesis; D. Pulse-feeding strategy. It stabilizes substrate influx during the production phase, reducing overflow metabolism and acetate accumulation compared with continuous feeding.

A representative quorum sensing (QS) circuit, shown in Fig. 2A, provides autonomous, cell-density-dependent regulation of pathway expression. Cells produce and release N-acyl homoserine lactones (AHLs), which accumulate as density increases, then re-enter cells to interact with regulators such as LuxR or EsaR [[21], [22], [23], [24]]. In a LuxR-type system, AHL binding activates the P_lux_ promoter, driving downstream gene transcription, while EsaR-based systems respond inversely to AHL concentration, enabling flexible control [[21], [22], [23], [24]].

This QS architecture has been successfully applied to decouple growth from production in multiple biosynthetic pathways. For l-homoserine production, Niu et al. introduced the esaI/esaR QS circuit from *Pantoea stewartii* into *E. coli* to dynamically downregulate *thrB* expression in a cell-density-dependent manner. During early fermentation, low AHL levels maintained *thrB* (encoding homoserine kinase)expression to support cellular growth; as cell density increased, AHL accumulated and relieved EsaR-mediated activation, thereby attenuating *thrB* transcription and redirecting carbon flux toward l-homoserine. This dynamic control enabled a final titer of 101.81 g/L l-homoserine with a yield of 0.41 g/g glucose in fed-batch fermentation [23]. In another study, Dinh and Prather engineered a bifunctional QS circuit combining LuxR and EsaR components to simultaneously up-regulate naringenin pathway genes (TAL and 4CL) and down-regulate competing reactions via CRISPRi, achieving a 140% increase in naringenin titer (463 μM) compared to static controls [22]. Similarly, Dai et al. implemented an EsaR-based QS system in *E. coli* to dynamically repress *sucA* (encoding the E1 subunit of 2-oxoglutarate dehydrogenase complex), thereby gradually shifting carbon flux from biomass formation to l-tyrosine biosynthesis. This strategy raised the cell-specific productivity to 0.048 g/g DCW/h, representing the highest reported spatiotemporal productivity for l-tyrosine production from glucose [24]. These examples illustrate the versatility of QS-based dynamic control: growth is prioritized during early stages, and production is activated only after a critical cell density is reached, thereby resolving the growth–production trade-off without exogenous inducers.

Beyond QS, other programmable systems (e.g., CRISPR interference [[25], [26], [27]], optogenetics [[28], [29], [30], [31]], and base-editing timers such as BioFuse [32,33]) have been developed for time-resolved control, each offering distinct trade-offs between reversibility, precision, and genetic stability. These observations highlight that practical application of dynamic control requires balancing regulatory precision, genetic stability, and process scalability; optimal performance is achieved by integrating genetic circuits with process-level regulation rather than by increasing control complexity alone.

### 2.2 Process-level phase segregation as an external temporal switch

Two-stage cultivation, also known as phase-segregated bioprocessing, separates cellular growth from product synthesis either spatially or temporally, thereby alleviating the inherent trade-off between biomass accumulation and metabolic burden [34]. Two-stage cultivation bioprocessing can be viewed as the process-level counterpart of intracellular dynamic control. Whereas QS, CRISPRi, or optogenetic circuits switch pathway expression from inside the cell, two-stage cultivation imposes the switch externally through nutrient, temperature, oxygen, or feeding regimes. In this approach, cells are initially cultivated under optimal growth conditions to maximize biomass formation. Once sufficient biomass is achieved, a controlled environmental change, such as nutrient limitation, carbon source switching, or a temperature shift, triggers a transition to the production phase, redirecting metabolic flux towards the target compound [35]. This approach is broadly applicable across batch, fed-batch, and continuous fermentation modes. For example, it has been successfully implemented in high-cell-density TRAIL production in *E. coli* and in lifespan-engineered yeast systems, where extending cellular viability sustains prolonged productivity [36,37].

As illustrated in Fig. 2B, the temporal separation results in distinct biomass accumulation and product formation phases, improving overall process efficiency and controllability. For complex biosynthetic pathways constrained by substrate competition, pathway imbalance, or intermediate toxicity, phase segregation can also be achieved at the population level. Sequential inoculation or co-culture strategies distribute pathway modules across different strains, reducing metabolic interference. For instance, a two-stage cultivation process for microbial lipid production first accumulates biomass under nitrogen-rich conditions, followed by a nitrogen-limited phase that triggers lipid accumulation, thereby temporally decoupling growth from product formation [38]. Such modular and temporal division of labor is particularly advantageous for the synthesis of structurally complex natural products.

A representative example of industrially relevant phase-segregated bioprocessing is the two-step biosynthesis of glycine from glucose. In this system, l-threonine is first produced via microbial fermentation, followed by its conversion to glycine through whole-cell catalysis. This temporally decoupled strategy achieved high titer and yield at the 50 L scale, demonstrating the scalability and practical feasibility of phase-separated production systems [39].

Overall, phase-segregated design provides a versatile framework for optimizing metabolic flux, wherein targeted arrest of cellular replication decouples growth from biosynthesis. Removal of the chromosomal origin of replication (*oriC*) via CRISPR–Cas9 irreversibly halts cell division while maintaining metabolic activity, redirecting resources to product synthesis (Fig. 2C). In *E. coli*, this strategy increased recombinant protein yields by 60% to 80% [40], with transcriptomic analysis showing a tenfold reduction in growth-associated gene expression and a 35% rise in ATP levels, reflecting reduced energy expenditure on replication. To preserve metabolic function during arrest, dual-chromosome designs maintain essential gene expression [40]. However, irreversible *oriC* excision limits application to batch processes where production follows biomass accumulation. Conditional systems such as temperature-sensitive variants enable reversible control, allowing dynamic growth–production transitions. Effective temporal decoupling requires balancing irreversibility, which offers high efficiency but low flexibility, with reversibility, which provides adaptability at greater complexity. No single strategy suffices to achieve efficiency by coordinating cellular states, enabling precise control over growth–production transitions, and enhancing yields in both simple and complex biosynthetic systems.

### 2.3 Temporal stabilization of substrate influx during production phase

Dynamic substrate delivery does not decouple growth and production in the strict sense of switching cells from a growth state to a production state. Instead, it supports temporal decoupling by stabilizing carbon influx during the production phase, preventing substrate overload, overflow metabolism, and stress responses that would otherwise force cells back into growth-associated or low-productivity metabolic states. Continuous substrate feeding frequently results in transiently elevated local substrate concentrations, which can exceed the processing capacity of central metabolic pathways (Fig. 2D). This overload saturates key glycolytic nodes, disrupts redox balance, and induces overflow metabolism, ultimately leading to the accumulation of inhibitory byproducts such as acetate [41]. The buildup of acetate not only reduces carbon efficiency but also imposes additional stress on cellular physiology, thereby limiting long-term productivity.

Dynamic pulse feeding offers an effective strategy to address this limitation. By intermittently supplying substrate in controlled pulses, carbon influx can be temporally aligned with the metabolic processing capacity of downstream pathways, particularly the tricarboxylic acid (TCA) cycle and the pentose phosphate pathway (PPP) [42]. This synchronization allows cells to fully assimilate incoming carbon before the next feeding event, preventing metabolic congestion and maintaining pathway balance. As a result, intracellular flux distributions remain stable, acetate overflow is minimized, and overall carbon utilization efficiency is significantly improved during prolonged fermentation processes [43].

Experimental studies further demonstrate the advantages of dynamic feeding strategies. In sorbose fermentation, intermittent pulsing of high-concentration sorbitol at 600 g/L triggered by dissolved oxygen signals, enhanced substrate uptake efficiency and improved productivity [44]. Similarly, adaptive feeding based on off-gas monitoring enabled real-time adjustment of substrate supply, increasing ethanol titers by 21% to reach 91 g/L compared with conventional fixed-rate feeding. Advanced control systems that integrate flux-oriented regulation further coordinate oxygen and substrate availability, enabling more precise metabolic control [45].

Under repeated substrate perturbations, microbial cells exhibit rapid metabolic adaptability. For instance, yeast cells maintained a glycolytic flux of up to 5.4 mmol/g/h without detectable accumulation of overflow metabolites under dynamic feeding conditions [46]. Thus, dynamic substrate delivery should be framed as an auxiliary temporal-control strategy: it does not itself create a growth-to-production switch, but it maintains the metabolic stability required for a decoupled production phase to operate efficiently.

### 2.4 Translational constraints and industrial feasibility

The successful implementation of these temporal strategies, and indeed all four approaches, in an industrial setting is contingent upon overcoming several key translational constraints. Plasmid burden and long-term genetic stability remain primary concerns; the constitutive expression of synthetic circuits during prolonged fed-batch fermentation can impose a significant drain on cellular resources, leading to genetic instability and mutation-driven loss-of-function. Population stability in microbial consortia is inherently challenging to maintain over many generations, as one subpopulation may outcompete the other, disrupting the engineered division of labor. Furthermore, scale-up reproducibility is fraught with difficulty due to non-ideal mixing, oxygen gradients, and pH heterogeneities present in large-scale bioreactors, which can unpredictably trigger the designed genetic switches or alter flux distributions. Finally, biocontainment of engineered strains and the challenges of engineering non-model host cells represent further barriers to widespread industrial adoption. Addressing these constraints will be a key focus for future work.

## 3 Network-level metabolic resource optimizations

Network-level metabolic reallocation addresses the growth–production trade-off by changing the allocation rules of the intracellular economy. In growing cells, carbon skeletons, ATP, NADPH, ribosomes, membrane capacity, and stress-response systems are preferentially used to support biomass formation [47]. Product synthesis competes with these same resources. Therefore, network-level strategies are relevant to the trade-off only when they redirect a limiting resource toward production while preserving enough cellular function to maintain viability and long-term productivity. Mechanistically, it comprises three interrelated dimensions, namely carbon flux redistribution, cofactor and energy balancing, and regulatory stabilization, each targeting distinct intervention layers within the metabolic network (Table 1).

**Table 1:** Comparative summary of representative mechanisms, performance outcomes, and trade-offs across temporal, metabolic, and spatial decoupling strategies.

Strategy	Target molecule	Benefits	Trade-offs/Limitations	Ref.
Temporal decoupling (two-phase fermentation with inducible promoter)	Isobutanol	2.5-fold increase in titer; complete separation of growth and production phases; reduced toxic intermediate interference during growth	Requires inducer addition (cost/leakage); genetic circuit instability over extended fermentation; residual growth in production phase reduces yield	[34]
Network-level resource optimization (PTS deletion + adaptive evolution)	L-Tyrosine	55% increase in yield; reduced acetate overflow; improved PEP availability for the shikimate pathway	∼20% reduction in growth rate; requires labor-intensive adaptive evolution to restore fitness; risk of reversion during scale-up	[48]
Network-level resource optimization (inducible NADPH-dependent HBD for butanol)	Butanol	+20% yield, +87.5% productivity; improved redox balance via temporally controlled cofactor supply	Inducible system required; risk of cofactor imbalance if induction timing is mis-optimized; inducer cost	[49]
Network-level resource optimization (Wss1-mediated DPCs repair for stress tolerance)	Cell growth (proxy)	3-fold improvement in growth under genotoxic stress; maintained genomic integrity; enhanced production phase viability	Pathway-specific (requires DNA damage stress); not applicable to non-stressed conditions; energy cost of repair machinery	[50]
Static spatial compartmentalization (SpyTag/SpyCatcher enzyme scaffold)	Resveratrol	5-fold titer increase; reduced intermediate (*p*-coumaroyl-CoA) accumulation; minimized cross-talk with competing pathways	Irreversible covalent assembly limits tunability; high protein burden from scaffold expression; cannot be disassembled for growth phase	[51]
Dynamic spatial compartmentalization (LLPS condensates via MED1 IDR)	Lycopene	2.8-fold increase; reversible clustering; tunable with inducer concentration; aligns with temporal separation	Condensate instability under high osmolarity or fluctuating pH (common during scale-up); ∼15% leakage of intermediates; incomplete isolation	[52]
AI-enabled dynamic control (feedback loop with biosensor + ML optimizer)	Violacein	3.1-fold improvement over static induction; real-time adaptation to metabolic state; reduced growth burden	Requires continuous online monitoring; model dependency (risk of overfitting); computational overhead; limited to well-characterized pathways	[53]

### 3.1 Carbon flux redistribution

**Fig. 3:**
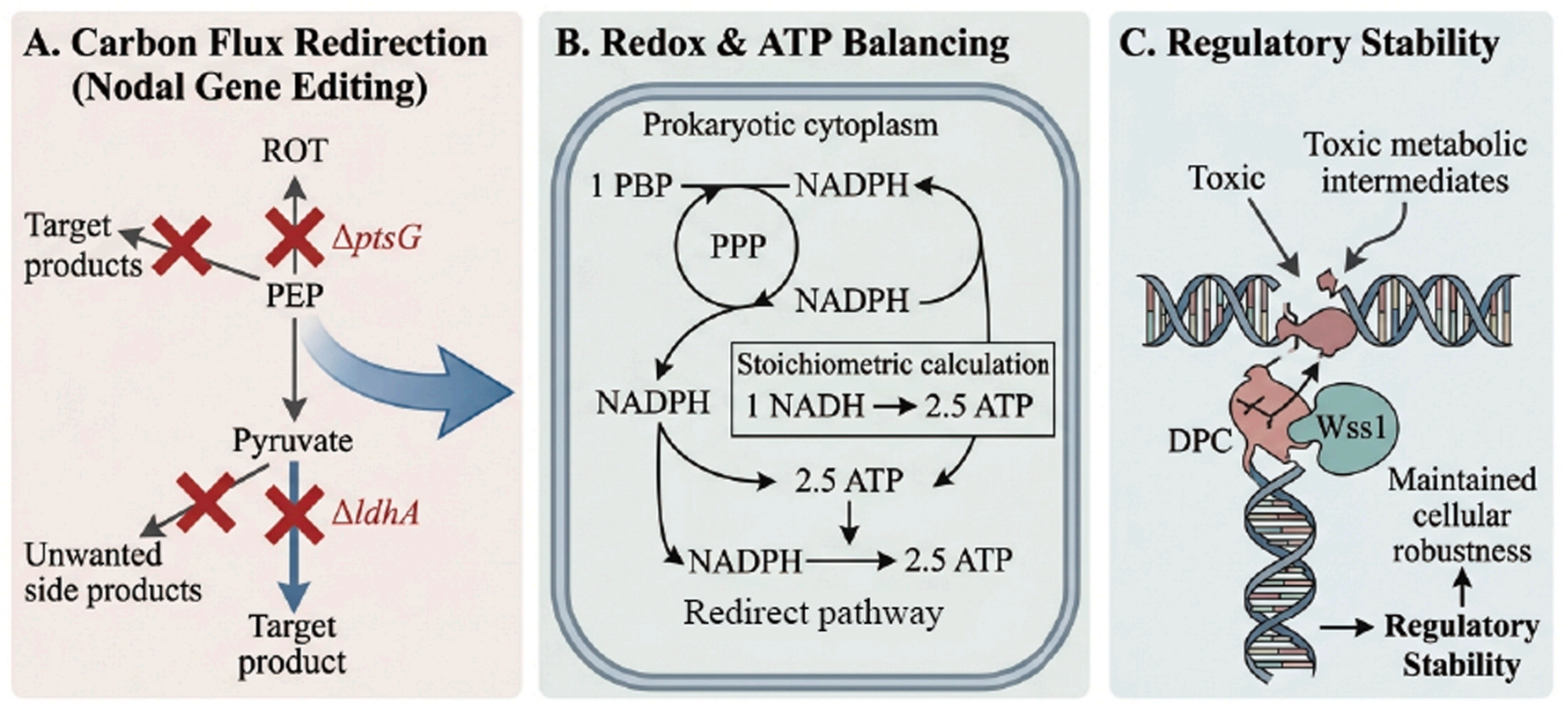
Network-level metabolic resource optimization strategies for redistributing carbon flux, energy, and regulatory capacity to balance growth and production in microbial cell factories. A. Carbon flux redirection via nodal gene editing at key metabolic branch points; B. Cofactor and energy balancing through coordinated NAD(P)H generation and ATP coupling; C. Regulatory stabilization through damage repair and stress-resilient network design.

Carbon flux redistribution aims to reallocate key precursor metabolites from biomass formation toward product synthesis. This is achieved by rewiring central metabolic nodes and substrate entry routes, thereby alleviating the intrinsic competition between cellular growth and bioproduction (Fig. 3A). Phosphoenolpyruvate (PEP) and pyruvate serve as critical branch points where carbon flux can be selectively redirected. By explicitly blocking competing pathways and preventing undesired byproduct formation while preserving productive routes, engineered systems enable the highly efficient channeling of carbon toward target molecules.

Nodal gene editing provides a direct and effective strategy to reshape these intracellular flux distributions. For example, the targeted deletion of phosphotransferase system (PTS) genes, such as glucose-specific PTS permease (*ptsG*) and PTS enzyme IIA component (*crr*), significantly reduces carbon consumption during glucose uptake while concurrently increasing PEP availability for downstream biosynthesis [48]. Similarly, the deliberate removal of competing metabolic pathways, including the deletion of lactate dehydrogenase A (*ldh*A), minimizes carbon loss to fermentation byproducts and enhances flux toward the desired end products. These targeted interventions function by simultaneously restricting carbon drainage and strengthening productive metabolic pathways at key junction points.

However, large-scale metabolic perturbations destabilize cellular homeostasis and constrain growth. While PTS-independent transport reconfiguration can relax native regulatory constraints [54], it imposes intrinsic trade-offs between carbon efficiency and growth, with excessive perturbation ultimately diminishing production performance, as observed in *Corynebacterium glutamicum* [55]. This suggests that carbon flux redistribution is limited by the global network architecture, necessitating coordinated control of upstream input and downstream capacity. Merely increasing local flux in isolation can induce downstream bottlenecks and may be inadequate to resolve the competition between growth and production. Carbon redistribution is effective only when the product pathway competes directly with biomass or byproduct-forming branches for the same precursor. If precursor redirection reduces growth too severely or creates downstream bottlenecks, the strategy shifts rather than resolves the trade-off.

### 3.2 Cofactor and energy balancing

Cofactor and energy balancing aligns NADPH and ATP supply with biosynthetic demand, determining pathway efficiency under growth-limited conditions (Fig. 3B) [56]. Even with proper carbon flux, insufficient NAD(P)H or ATP often limits production.

Redox engineering enhances reducing power generation and utilization. Upregulating NADPH-producing routes boosts biosynthesis [57,58]; introducing NADPH-dependent enzymes improves butanol production [49,59,60]. Reinforcing NAD(P)H and ATP also enhances substrate utilization [61]. To resolve ATP competition between growth and production, orthogonal ATP regeneration systems provide an independent energy supply [62,63].

However, redox or ATP perturbations can impact growth and stress responses [64]. Cofactor and ATP engineering resolves the trade-off when production is limited by NADPH, NADH, or ATP demand that normally supports growth and maintenance. Thus, cofactor strategies require coordinated regulation and are most effective when production is directly limited by reducing power or ATP supply.

### 3.3 Regulatory stability and cellular robustness

Regulatory stability relies on maintaining cellular functionality during periods of intensified metabolic flux, resource redistribution, and the accumulation of potentially toxic intermediates. Elevated production states frequently cause molecular damage and regulatory disruption, such as DNA-protein cross-links (DPCs) alongside severe transcriptional interference, which ultimately compromise cellular robustness (Fig. 3C).

Integrating non-metabolic support systems offers an effective strategy to mitigate these challenges. For instance, deploying targeted repair mechanisms for DPCs damage, such as the Wss1 protease system, significantly enhances cellular tolerance to toxic intermediates and preserves genomic integrity under stress conditions [50]. By ensuring continuous DNA accessibility and stable transcriptional activity, these repair systems facilitate sustained metabolic function throughout prolonged production phases.

Furthermore, the rational refactoring of regulatory circuits improves the predictability and stability of overall gene expression. Tunable regulatory architectures, including engineered two-component systems (TCS), permit the independent control of signal sensing and subsequent response outputs [65]. This functional decoupling reduces regulatory noise, improves system responsiveness, and stabilizes pathway expression amidst fluctuating environmental and metabolic conditions.

Collectively, regulatory stabilization strategies supply the essential structural and functional foundations required for long-term bioproduction stability. Successful metabolic resource optimization relies not only on the efficient redistribution of cellular resources but also on the inherent capacity of the host cells to maintain functional integrity under engineered stress. The robustness engineering does not directly increase carbon flux, but it expands the cellular tolerance window in which high-flux production can be maintained without collapse of transcription, protein quality control, or genome stability.

### 3.4 Design principles and system-level insights

The four principles outlined above are not merely conceptual hypotheses but are distilled from and substantiated by a growing body of experimental evidence. The principle that carbon flux redistribution must be coordinated at the network level is validated by Tian et al. [48], where *ptsG* and *crr* deletion successfully redirected PEP flux only when coupled with biosensor-guided evolution to restore growth. Conversely, Ou et al. [55] provide a clear counterexample in *C. glutamicum*, demonstrating that excessive, uncoordinated perturbation (PTS deletion) can severely compromise titer, thereby underscoring the necessity of system-level balance. The principle of aligning cofactor and energy supply is supported by the inducible NADPH-dependent 3-hydroxybutyryl-CoA dehydrogenase (HBD) strategy for butanol production [49] and the orthogonal ATP regeneration system [63], both of which show that cofactor engineering can resolve the trade-off when a specific cofactor is limiting. Finally, the principle of regulatory robustness is directly illustrated by the Wss1-mediated DPCs repair system [50], which preserved genomic integrity under stress, and by the rationally refactored TCS [65], which improved signal–response stability. Together, these examples substantiate the assertion that metabolic resource optimization is a system-level coordination problem whose success depends on simultaneous optimization across multiple, interconnected dimensions.

## 4 Spatial reorganizations to isolate metabolic conflict and toxicity

**Fig. 4:**
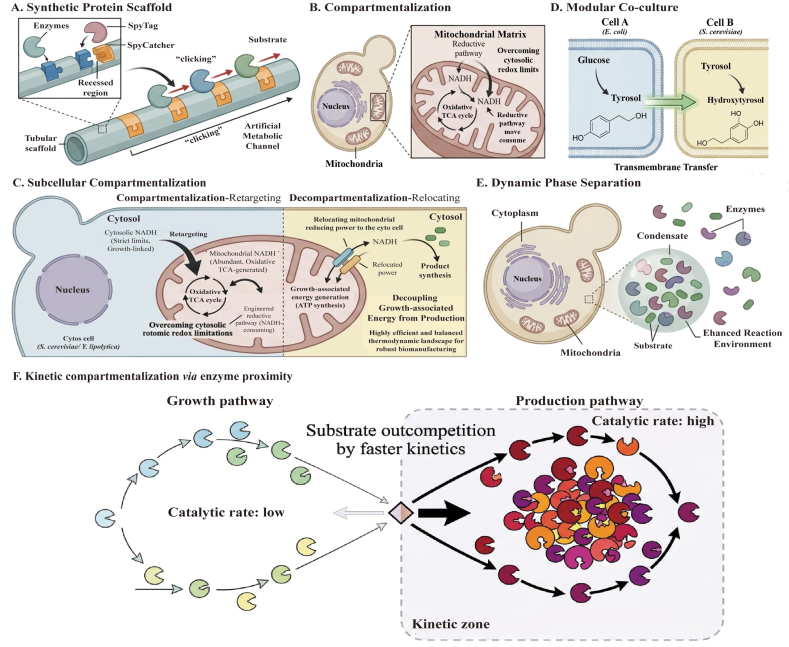
**Multi-scale spatial reorganization strategies for decoupling metabolic flux and alleviating growth–production trade-off in microbial cell factories.** A. Synthetic protein scaffolds for enzyme colocalization and substrate channeling (SpyTag/SpyCatcher system); B. Organelle-based compartmentalization for redox coupling and metabolic specialization (mitochondrial targeting); C. Subcellular compartmentalization engineering via retargeting and decompartmentalization to redistribute cofactors and decouple growth from production; D. Modular co-culture systems enabling intercellular division of labor and pathway partitioning; E. Dynamic phase separation for forming transient metabolic condensates and enhancing local reaction environments; F. A production pathway with high local enzyme concentration and catalytic rate outcompetes the growth pathway for a shared intermediate, achieving functional isolation without physical barriers.

Spatial reorganization strategies mitigate the growth–production trade-off by physically or kinetically separating competing metabolic processes across scales (Fig. 4). By isolating metabolic flux within defined domains, these approaches enhance local reaction rates, reduce intermediate toxicity, and minimize cross-pathway interference.

### 4.1 Subcellular compartmentalization for metabolic isolation

#### 4.1.1 Static compartmentalization: enzyme scaffolds and organelles

At the molecular scale, enzyme colocalization via synthetic scaffolds (e.g., SpyTag/SpyCatcher) creates irreversible covalent metabolic channels, enabling substrate channeling and reducing intermediate loss [51,66]. This approach protects labile intermediates and improves pathway flux (Fig. 4A) [67]. At the organelle level, mitochondrial compartmentalization exploits localized cofactor/redox pools to enhance reductive biosynthesis; decompartmentalization can redistribute reducing equivalents to the cytosol, decoupling growth from production (Fig. 4B and C) [68,69]. Static strategies offer high stability but lack adaptability—once assembled, they cannot be easily disassembled or tuned.

#### 4.1.2 Dynamic compartmentalization: biomolecular condensates

Liquid–liquid phase separation (LLPS) forms reversible, membraneless condensates that concentrate enzymes and substrates (Fig. 4E) [70]. Their rapid assembly/disassembly enables dynamic flux control, aligning with temporal decoupling. However, LLPS is sensitive to industrial conditions (pH, temperature, osmolarity) and provides incomplete isolation, making it best suited for stimulus-responsive pathways [70]. The choice between static and dynamic compartmentalization depends on required stability vs. flexibility. Future hybrid designs may combine strengths of both paradigms.

### 4.2 Functional specialization among cells

At the population level, spatial separation can be engineered by partitioning complex metabolic pathways across distinct microbial strains (Fig. 4D). This modular architecture segregates upstream and downstream reactions into separate cellular environments, alleviating metabolic burden on individual cells and allowing each constituent strain to operate under its respective optimal conditions. Furthermore, this division of labor enables functional integration of incompatible metabolic processes that cannot readily coexist within a single host [52,71,72].

A prominent example is the co-culture system in which *E. coli* converts glucose into tyrosol; this intermediate is subsequently transported across the cell membrane and further metabolized into hydroxytyrosol by *Saccharomyces cerevisiae* (Fig. 4B). Similarly, distributing pathway steps across species boundaries has facilitated efficient *de novo* biosynthesis of tryptophol from glucose using a modular *E. coli*–*S. cerevisiae* consortium [53]. However, as indicated by the obligatory transmembrane transfer of metabolites (Fig. 4B), overall performance is intrinsically bottlenecked by intermediate exchange efficiency and maintenance of balanced population dynamics. The success of intercellular strategies hinges on stable synergistic interactions and coordinated metabolite flux, rather than solely on optimization of individual strains.

### 4.3 Kinetic compartmentalization: a new frontier

Kinetic compartmentalization achieves functional isolation without physical enclosures, exploiting reaction kinetics and enzyme proximity [73]. When two pathways share an intermediate, the pathway with higher local enzyme concentration and faster catalysis can kinetically outcompete the other, creating a transient substrate-depleted zone. This can be realized through enzyme proximity effects (e.g., fusion proteins, short-range scaffolds) or substrate channeling via electrostatic tunnels or flexible linkers [74]. Its key advantage is minimal metabolic burden and self-regulation; limitations include strong dependence on enzyme kinetic parameters and inability to sequester toxic intermediates (Fig. 4F).

### 4.4 Summary design principles

Across scales, spatial reorganization operates by isolating metabolic flux within defined domains: proximity promotes substrate channeling [51,66]; separation minimizes interference between native and engineered pathways; compartment-specific environments can be tailored to match pathway requirements [68,69]; and isolation must be balanced with transport, as excessive confinement introduces new limitations [70]. Together, these principles transform metabolic interference from an inherent challenge into a controllable design parameter.

## 5 AI-enabled control systems for microbial cell factories

**Fig. 5:**
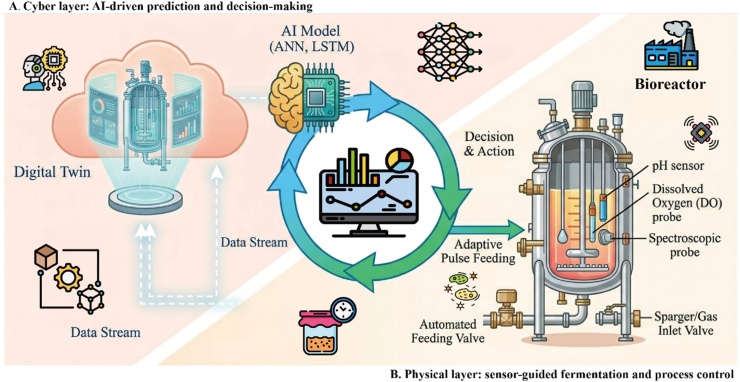
**AI-enabled cyber–physical framework for adaptive biomanufacturing.** A. Cyber layer: AI models and digital twins analyze real-time fermentation data and predict system states to support decision-making; B. Physical layer: Bioreactor sensors continuously monitor process variables, while automated actuators implement adaptive feeding and control strategies. Real-time process data collected from bioreactor sensors (e.g., pH, dissolved oxygen, and spectroscopic probes) are transmitted to the cyber layer, where AI models and digital twins analyze system states and predict fermentation performance. The resulting control decisions are fed back to the physical layer through automated actuators, enabling adaptive pulse feeding and closed-loop optimization of microbial production processes.

Digital and intelligent control frameworks introduce a cyber-physical regulatory layer that connects computational models with real-time bioprocess operation, enabling closed-loop optimization of cell factory performance (Fig. 5, Table 2) [75].

**Table 2:** AI–enabled frameworks supporting multi-scale decoupling and process optimization in food biomanufacturing.

Decoupling dimension	Core challenge	AI tools	Mechanistic contribution	Ref.
Temporal	Optimal switching between growth and production	ANN; RNN/LSTM; soft sensors	Time-series prediction, delay modeling, and real-time state estimation for closed-loop control	[[76], [77], [78], [79], [80]]
Metabolic	Preferential allocation of carbon and energy to growth	ANN–GA; FNN; PSO	Multi-parameter optimization and high-dimensional flux search	[[81], [82], [83], [84], [85], [86]]
Spatial	Bioreactor heterogeneity and mass-transfer limits	CNN; IoT; ANN control	Spatial feature extraction and localized adaptive control	[[87], [88], [89]]
Data scarcity	Limited and noisy fermentation datasets	GANs; data augmentation	Synthetic data generation and improved generalization	[89,90]
Interpretable dynamic modeling	Nonlinear and time-varying metabolic interactions	Hybrid DNN–mechanistic models; PINNs	Mechanism-informed prediction and pathway coupling reconstruction	[[91], [92], [93]]
System-level integration	Lack of predictive, adaptive manufacturing control	Digital twin frameworks	Virtual–physical mapping and unified multi-scale optimization	[94]

### 5.1 Data-driven prediction, optimization, and cyber-physical control

During fermentation, AI models analyze real-time sensor data to predict system states. Neural networks estimate product formation and substrate consumption without requiring fully defined mechanistic models [76]; recurrent neural networks (RNN) and long short-term memory (LSTM) networks capture temporal dependencies in fermentation dynamics, enabling accurate prediction of nonlinear behaviors [77]; soft sensors infer difficult-to-measure variables (e.g., biomass concentration) from readily accessible signals including pH, dissolved oxygen (DO), and spectroscopic data [[78], [79], [80]].

Predicted states guide control decisions. For example, artificial neural networks combined with genetic algorithms optimize medium composition and induction strategies to enhance flux toward target products [[81], [82], [83], [84]]; fuzzy neural networks improve control stability under highly coupled metabolic conditions [85]. A more specific illustration is particle swarm optimization (PSO)-driven adaptive pulse feeding: the system dynamically adjusts substrate feed rates based on predicted metabolic capacity. In an industrial case of sorbose fermentation, intermittent pulses of high-concentration sorbitol (600 g/L) triggered by DO signals significantly enhanced substrate uptake efficiency and productivity. In ethanol fermentation, adaptive feeding based on off-gas monitoring increased ethanol titer by 21% to reach 91 g/L, a level unattainable with conventional constant-rate feeding. This strategy prevents overflow metabolism (e.g., acetate accumulation) caused by substrate overload, thereby improving process stability [86].

The integration of AI with the Internet of Things (IoT) enables real-time coupling between the cyber (Fig. 5A) and physical layers (Fig. 5B). Sensors embedded in the bioreactor—pH probes, DO sensors, and spectroscopic devices—continuously monitor process conditions. Data streams are transmitted to computational models that interpret system states and generate control actions. Convolutional neural networks (CNN) can further analyze spectral or imaging data to resolve spatial heterogeneity (e.g., local substrate gradients) during fermentation [87]. Actuators (feeding valves, gas flow control systems) are adjusted accordingly, establishing a closed-loop cyber-physical system that reduces batch-to-batch variability and enhances reproducibility [88].

### 5.2 Hybrid modeling, digital twins, and autonomous biomanufacturing

Hybrid models and digital twins do not directly resolve the growth–production trade-off by introducing new metabolic pathways. Instead, their primary value lies in anticipating when cells approach trade-off dominated states, such as substrate overflow, oxygen limitation, redox imbalance, or productivity collapse, and dynamically adjusting feeding, induction, aeration, or temperature to maintain cells within a productive physiological window. To address the limitations of purely data-driven or purely mechanistic models, hybrid modeling frameworks integrate both approaches. Mechanistic models provide interpretability and physical constraints, while machine learning captures complex nonlinear relationships. For example, generative adversarial networks (GANs) generate synthetic training datasets to enhance model robustness under sparse or noisy conditions [89,90]. Hybrid models combining kinetic equations with neural networks improve prediction of fermentation dynamics [91,92]. Physics-informed neural networks (PINNs) embed biochemical constraints into the learning process, increasing reliability under uncertainty [93].

A digital twin functions as a virtual replica of the bioreactor that continuously updates with real-time process data, enabling simultaneous simulation, prediction, and optimization alongside the physical system. This bidirectional coupling allows online validation of model predictions and adaptive tuning of process conditions. Digital twins support autonomous control strategies in which the system iteratively optimizes performance through continuous learning and feedback. This framework has been successfully applied to the production of high-value compounds, such as polyphenols and human milk oligosaccharides from agricultural feedstocks. In these applications, the digital twin predicts product titers and substrate consumption in real time and automatically adjusts feed rates and induction timing to significantly improve final yield and process consistency [94]. Therefore, digital twins should be regarded as an enabling layer for trade-off management rather than as an independent biological solution. Their effectiveness depends on whether model predictions are explicitly linked to controllable variables that influence resource allocation, including substrate uptake rate, induction timing, oxygen transfer, and growth-arrest scheduling.

## 6 Conclusion and perspectives

Although all four strategies aim to resolve the growth–production trade-off, each is optimally suited to specific scenarios. Temporal decoupling is most effective when achieving high biomass is essential before product synthesis and when production can tolerate a delayed onset; however, it is constrained by the metabolic burden and potential instability of synthetic genetic circuits.

Network-level reallocation excels when the target product competes directly for a well-defined precursor, but excessive network perturbation may compromise cellular fitness. Spatial reorganization is particularly advantageous for pathways that are toxic or involve multiple sequential steps causing intracellular interference, with the central challenge being the balance between efficient metabolite transport and effective isolation. AI-enabled control provides an overarching, adaptive layer capable of optimizing the other three strategies; its limitation lies in reliance on high-quality real-time data and the robustness of predictive models under heterogeneous industrial conditions. Addressing these limitations requires convergent technological advances.

Hybrid modeling that integrates mechanistic understanding with machine learning can improve predictive accuracy under variable conditions. Modular genetic circuit architectures with minimal metabolic overhead are necessary to ensure long-term stability. Together, these integrated innovations will enable next-generation microbial cell factories that are both productive and resilient, fully realizing the potential of spatiotemporal decoupling for sustainable biomanufacturing.

## Funding sources

This work was financially supported by the 10.13039/501100001809National Natural Science Foundation of China (Grant No. 32272287) and the 10.13039/501100012166National Key Research and Development Program of China (Grant No. 2022YFD1802104).

## CRediT authorship contribution statement

**Huanghui Xia:** Visualization, Writing – original draft, Writing – review & editing. **Bingmei Wang:** Supervision. **Feng Qi:** Funding acquisition, Supervision, Writing – original draft, Writing – review & editing. **Jianzhong Huang:** Supervision.

## Declaration of competing interest

The authors declare no competing financial, personal, or professional interests that could have influenced the work reported in this manuscript. No funding sources or sponsors have influenced the design, execution, interpretation, or publication of this study. The authors alone are responsible for the content and writing of this paper.

## ABBREVIATIONS

4CL, 4-coumarate:CoA ligase; AHLs, N-acyl homoserine lactones; AI, artificial intelligence; ANN, artificial neural network; ATP, adenosine triphosphate; CNN, convolutional neural network; CPS, cyber-physical system; CRISPRi, CRISPR interference; DO, dissolved oxygen; DPCs, DNA-protein cross-links; FNN, fuzzy neural network; GAN, generative adversarial network; GRAS, generally recognized as safe (U.S. FDA designation); HBD, 3-hydroxybutyryl-CoA dehydrogenase; IoT, Internet of Things; LSTM, long short-term memory; ML, machine learning; NADH, reduced nicotinamide adenine dinucleotide; NADPH, reduced nicotinamide adenine dinucleotide phosphate; oriC, chromosomal origin of replication; PEP, phosphoenolpyruvate; PINN, physics-informed neural network; PPP, pentose phosphate pathway; PSO, particle swarm optimization; PTS, phosphotransferase system; QS, quorum sensing; RNN, recurrent neural network; TAL, tyrosine ammonia-lyase; TCA, tricarboxylic acid cycle; TCS, two-component systems.
